# Rat model of asphyxia-induced cardiac arrest and resuscitation

**DOI:** 10.3389/fnins.2022.1087725

**Published:** 2023-01-04

**Authors:** Shuhang Yu, Chenghao Wu, Ying Zhu, Mengyuan Diao, Wei Hu

**Affiliations:** Department of Critical Care Medicine, Affiliated Hangzhou First People’s Hospital, Zhejiang University School of Medicine, Hangzhou, China

**Keywords:** cardiac arrest, asphyxia, experimental animal models, rat, neurological deficit

## Abstract

Neurologic injury after cardiopulmonary resuscitation is the main cause of the low survival rate and poor quality of life among patients who have experienced cardiac arrest. In the United States, as the American Heart Association reported, emergency medical services respond to more than 347,000 adults and more than 7,000 children with out-of-hospital cardiac arrest each year. In-hospital cardiac arrest is estimated to occur in 9.7 per 1,000 adult cardiac arrests and 2.7 pediatric events per 1,000 hospitalizations. Yet the pathophysiological mechanisms of this injury remain unclear. Experimental animal models are valuable for exploring the etiologies and mechanisms of diseases and their interventions. In this review, we summarize how to establish a standardized rat model of asphyxia-induced cardiac arrest. There are four key focal areas: (1) selection of animal species; (2) factors to consider during modeling; (3) intervention management after return of spontaneous circulation; and (4) evaluation of neurologic function. The aim was to simplify a complex animal model, toward clarifying cardiac arrest pathophysiological processes. It also aimed to help standardize model establishment, toward facilitating experiment homogenization, convenient interexperimental comparisons, and translation of experimental results to clinical application.

## 1. Introduction

Since the development of cardiopulmonary resuscitation (CPR), an increasing number of patients now have return of spontaneous circulation (ROSC) and are discharged from the hospital. The survival rate among patients who have had cardiac arrest (CA) has increased by ∼10% (Yamaguchi et al., 2017). However, ROSC is merely the first step in successful treatment, as many surviving patients live with neurologic deficits that lead to poor outcomes (Bougouin et al., 2014). This is due to the systemic tissue ischemia and hypoxia caused by CA, which can lead to post-resuscitation reperfusion injury (Eltzschig and Eckle, 2011). Moreover, post-CA hypoxic ischemic brain injury is the leading cause of death and poor long-term outcomes (Sekhon et al., 2017). We now face the clinical challenge of improving post-resuscitation outcomes through early assessment of neurologic deficits. To this end, we require complex animal models to determine how we can modify CA-induced pathophysiological processes. A systematic review about contemporary animal model of CA reported that there existed multiple CA animal models, but the models’ great heterogeneity along with great variability in definitions and reporting make comparisons between studies difficult (Vognsen et al., 2017). In basic science studies, there is often a lack of reproducibility between laboratories, for the differences in animal species, protocols, type of anesthesia, and so on (Granfeldt, 2016). In addition, most animal models do not truly reflect clinical pathophysiological processes, such as lacking comorbidities or post-resuscitation management (Granfeldt, 2016). These limitations prevent the results of animal models from being applied to human neurologic protection guidelines. Standardized models enable more homogenous experiments, allow convenient comparisons between experiments, and facilitate translation of experimental results to clinical applications.

Over the past 30 years, the etiology of CA has shifted from sudden CA (SCA) to asphyxia-induced CA (ACA) (Dezfulian and Lavonas, 2020; Van den Bempt et al., 2021). SCA refers to sudden cardiac output cessation, characterized by ventricular fibrillation (VF) or pulseless ventricular tachycardia (Zimmerman and Tan, 2021). In contrast, ACA refers to breath cessation and presents as pulseless electrical activity (PEA), leading to progressive hypoxia and eventual CA. There are several differences between ACA and SCA regarding post-resuscitation neurologic deficits. From the pathophysiology perspective (Dezfulian and Lavonas, 2020), anoxia perfusion plays a dominant role in ACA-related brain injury. Anoxia perfusion exacerbates lactic acidosis and leads to free radical injury, which contributes to brain injury (Dezfulian and Lavonas, 2020). Distinct from ACA, during SCA anoxic perfusion is absent because circulation ceases (Dezfulian and Lavonas, 2020). This accounts for the greater severity of brain injury in ACA compared with SCA. A systematic review reported that about 54% of CA animal studies model SCA (VF-induced CA), compared with only 25% that model ACA (Vognsen et al., 2017). Thus, this review summarizes the procedures for establishing an ACA animal model, to provide a standardized model for future CA research.

## 2. Attributes of rats for modeling ACA

We need to establish a standard ACA model toward developing effective CA diagnostics, treatments, and prevention measures. Several attributes make the rat an ideal model for CA research (Table 1). Rats and humans have similar hemodynamic parameters during resuscitation (Barouxis et al., 2012). Excepting that the rat heart rate is significantly faster, other hemodynamic parameters like mean arterial pressure (MAP), right atrial pressure, coronary perfusion pressure (CPP) are similar (Popp et al., 2007). Financially, rats confer cost-based advantages compared with other mammals like rabbits and swine (Reid et al., 2003). Moreover, a rat model can be operated alone. Transgenic rat technology and *in vitro* rat cell line have also matured, allowing more basic CA investigations. Compared with mice, rats have greater blood and tissue volumes for meeting testing requirements. Furthermore, standardized assessments of rats have been developed to evaluate neurologic deficits, [e.g., the neurologic deficit score (NDS), described below]. Finally, we have a thorough understanding of rodent anatomy and physiology, facilitating extrapolation of study findings to humans (Diao et al., 2020).

**TABLE 1:** Comparison of different animal cardiac arrest (CA) models.

	Advantages	Disadvantages
Rat	The hemodynamic index is similar to that of human. Sufficient tissue for subsequent testing. Low price and easy to feed. Experiment can be operated alone. *In vitro* rat nerve cell lines are mature.	Heart rate is different from that of humans. Small animals have difficulty to operate (but easier than mouse relatively).
Mouse	The hemodynamic index is similar to that of human. Low price and easy to feed. *In vitro* mouse nerve cell lines are mature.	Heart rate is different from that of humans. Insufficient tissue for subsequent testing. Small animals have difficulty to operate.
Rabbit	The hemodynamic index is similar to that of human. Sufficient tissue for subsequent testing. Low price.	The cardiovascular structure and physiology differ greatly from that of human. Require at least two operators to cooperate. *In vitro* rabbit nerve cell lines are immature.
Swine	Physiology and anatomy are similar to humans. Sufficient tissue for subsequent testing.	Expensive price. Large animals require several operators to cooperate. The few antibodies designed for swine limit the subsequent testing. *In vitro* swine nerve cell lines are immature.

Different animal CA models’ advantages and disadvantages are listed.

After selecting rats as experimental animals, we should consider the rats’ type, sex, weight, and age of the rats. Reading recent literature, Sprague Dawley rats (Chen et al., 2019; Diao et al., 2020) were widely employed, while Wistar rats (Kuklin et al., 2019) and Long-Evans rats (Kim et al., 2016) were also used. These three types of rats are cultured from the outbred strain, hence, the individual heterogeneity between rats can reflect the heterogeneity of the CA patient to some extent. Human epidemiological studies have shown that the CA characteristics are different in men and women, and there is a correlation between women and increased survival rates (Wissenberg et al., 2014). In the current research, Studies favored males over females to represent the whole population, which is obviously biased. Therefore, in the experimental CA animal study, the gender differences and its underlying pathophysiological mechanism may also be an important research yield. When it comes to rats’ age, it depends on the purpose of the study (Vognsen et al., 2017). Normally, we choose healthy adult rats (2–18 months) as model animals. Taking feeding costs into account, rats aged 2–3 months are mostly applied in the literature. Pediatric rats or aged rats are also applied in experiments on newborn and elderly populations. Under reasonable feeding conditions, the body weight of rats was related to age to a certain extent. Rats aged 2–3 months can generally reach 250–450 g. What we tend to overlook here is that the majority of CA patients are not healthy, they are usually complicated with organic disease. A study of hypertensive CA rats showed that hypertension groups got more severe brain damage and lower survival than the control group (Wang et al., 2016). It reflected differences between healthy CA patients and CA patients with other co-morbidities. This may affect the translation of animal results into the clinic. However, there are few studies on the comorbidity model. It is necessary to develop the comorbidity model in the future.

In summary, we support that rats have potential as ACA animal models and that the selection of specific rat age and sex needs to be matched to the purpose of the study.

## 3. Factors to consider in establishing an ACA rat model

### 3.1. Anesthesia methods

Anesthesia, the first step in establishing a standardized ACA rat model, includes induction, maintenance, and withdrawal. In most cases, anesthesia induction in small animals is carried out using narcotic gases (Cicero et al., 2018) like isoflurane, sevoflurane, halothane, or carbon dioxide (CO_2_), which are usually released in a small, enclosed space (Lee et al., 2017b). Anesthesia can reduce the animal’s stress response and ease surgical procedures like endotracheal intubation and intraperitoneal administration.

According to the recent literature, pentobarbital, sevoflurane, isoflurane, and chloral hydrate are most commonly used in research to maintain anesthesia (Table 2). Maintenance of a certain anesthesia depth is necessary for surgery, to avoid the influence of increased catecholamines on brain metabolism (Gough and Nolan, 2018). Anesthesia can be maintained by micropump injection or scheduled administration. Supplemental drugs can be added by observing the heart rate, blood pressure, and response to painful stimuli (e.g., tail clipping stimulation).

**TABLE 2:** Modeling factors to consider.

	Anesthesia method	Neuromuscular blocking agent	Definition of CA	Duration of CA	Definition of ROSC	Definition of failed ROSC
Tungalag et al., 2022	2–3% isoflurane by inhalation	Vecuronium, 2 mg/kg	MAP < 20 mmHg	5 min	MAP ≥ 60 mmHg	Not mentioned
Zhang et al., 2022	Isoflurane by inhalation (4% induction, 1–3% maintenance)	Vecuronium, 2 mg/kg	MAP < 20 mmHg	8 min	MAP ≥ 60 mmHg lasting for ≥ 5 min	Duration of CPR > 5 min
Uray et al., 2021	Isoflurane by inhalation (4% induction, 2% maintenance)	Cisatracurium 1 mg/kg	MAP < 10 mmHg	10 min	Sustained supraventricular rhythm with MAP > 50 mmHg	Duration of CPR > 4 min
Hu et al., 2019	Pentobarbital by intraperitoneal injection (45 mg/kg induction, 10 mg/kg/h maintenance).	Pipecuronium, 0.1 mg/kg	MAP < 30 mmHg	7 min	MAP ≥ 60 mmHg lasting for ≥ 5 min	Duration of CPR > 10 min
Zhou et al., 2017	10% chloral hydrate by intraperitoneal injection (0.3 ml/100 g)	No	MAP < 25 mmHg	5 min	MAP ≥ 60 mmHg lasting for ≥ 10 min	Not mentioned
Keilhoff et al., 2017	5% sevoflurane	Vecuronium, 1 mg/kg	MAP < 10 mmHg	6 min	MAP ≥ 60 mmHg	Duration of CPR > 2 min

Select, recent papers illustrating different modeling methods. CA, cardiac arrest; ROSC, return of spontaneous circulation; MAP, mean arterial pressure.

However, analgesia was neglected in many experiments. This does not meet the ethical and regulatory requirements of maximizing animal welfare (Carbone and Austin, 2016). Therefore, we suggest that analgesia should be added to the process of animal anesthesia. Buprenorphine, carprofen, and meloxicam are commonly used analgesics in rats. When planning the timing of administration, the onset time of analgesic drug must be taken into account. Compared with oral drugs, subcutaneous and intraperitoneal administration takes 15–30 min to take effect. The subcutaneous injection can avoid damage to abdominal organs (Herrmann and Flecknell, 2019). Therefore, the subcutaneous injection may be a better mode of drug administration. The recommended doses of buprenorphine, carprofen, and meloxicam were 0.01–0.1, 2–5, and 1–2 mg/kg, respectively, by subcutaneous injection (Foley et al., 2019).

Anesthesia should be withdrawn before CA is induced, to reduce its cardiovascular impacts. Rats show reduced O_2_ saturation and heart rate after administration of an anesthetic mixture (Kirihara et al., 2016). Murakami et al. compared inhalation (isoflurane) and intraperitoneal injection (pentobarbital) anesthesia, showing that both decreased heart rate and blood pressure in rats. Since pentobarbital has a more obvious effect on heart rate and blood pressure, isoflurane is recommended from the hemodynamic perspective (Murakami et al., 2014). Therefore, CA should be induced when the rat is about to recover from anesthesia, reducing the effects of anesthetics on resuscitation outcomes and homogenizing the experimental model.

Our team’s procedure is to use CO_2_ to induce anesthesia, and then inject 3% sodium pentobarbital intraperitoneally for maintenance at a dose of 45 mg/kg (Diao et al., 2020). The first dose usually maintains for about 1 h. Additional doses of 0.1 ml of 3% sodium pentobarbital are then used to maintain anesthesia for ∼30 min–1 h. CA is begun following anesthesia washout.

### 3.2. Endotracheal intubation

Endotracheal intubation is challenging in rat experiments for the difficulty to see the epiglottis and vocal cords. 14G venous catheter (45 mm length) is often used for endotracheal catheter (Foley et al., 2019). The classical blind oral intubation requires the operator’s proficiency (Stark et al., 1981). Repeated intubation attempts can lead to laryngeal edema, glottic injury, and death from respiratory failure. In recent years, tracheal intubation under a visual laryngoscope has been realized, and the intubation efficiency is higher (Balzer et al., 2020). However, for laboratories without advanced facilities, endotracheal intubation can be performed on the premise of exposing the neck. In the case of looking directly at the trachea, to some extent, it can assist with endotracheal intubation (Diao et al., 2020).

### 3.3. CA induction method—asphyxiation

Asphyxia cardiac arrest is usually induced by disconnecting the mechanical ventilator and clamping the tracheal tube, with or without vecuronium. In recent years, the ACA model has also been used for *in vitro* cardiopulmonary cerebral resuscitation studies (Wollborn et al., 2019; Yin et al., 2021). It can accurately model the main causes of CA and death due to asphyxia, including post-CA changes in blood gases and pathophysiology of the heart, brain, kidney, and other tissues. This method requires simple equipment and procedures. Because it does not require thoracotomy, it has minimal effects on lung function.

Even under sedation, rats struggle when ACA is induced, which may cause equipment disconnection that may affect the experiment. Vecuronium can prevent this by reducing animal sensitivity. A neuromuscular blocking agent, it is administered to induce apnea without adverse cardiovascular effects (Jurado and Gulbis, 2011). A muscle relaxant is used first to reduce the stress response, with which apnea time tends to be consistent. Other muscle relaxants like pecuronium, cisatracurium, or rocuronium are also used in rat models (Table 2).

Our procedure is to administer vecuronium at a dose of 2 mg/kg by intravenous injection, leading to an apnea duration in rats of ∼15 s (Diao et al., 2020).

### 3.4. CA duration

Operationalized CA duration varies among investigators. Some define this as lasting from the start of disconnecting the mechanical ventilator to clamping the tracheal tube, while others define it as the no-flow period (i.e., no perfusion, which causes the MAP decrease to a certain value, often 25 or 20 mmHg depending on the experimental design) (Table 2).

Our procedure is to divide asphyxiation into three phases. Phase 1 is from clamping the tracheal tube to apnea, which generally lasts 15 s with vecuronium. Phase 2 is hypoxic perfusion, when apnea occurs with some perfusion, generally lasting 3 min. Phase 3 is no-flow, with duration depending on the experimental design. It can be inferred that prolonging CA duration will aggravate neurologic deficits and decrease the ROSC rate. The recent literature indicates that CA duration usually ranges from 4 to 10 min. In our experience, a 6-min CA model achieves neurologic deficits while maintaining a high ROSC rate (>80%), while use of a 7-min CA model reduces the ROSC rate to ∼50%.

### 3.5. CPR

It is well known that high-quality CPR plays an important role in improving CA survival. Standard CPR procedures include chest compressions, mechanical ventilation, epinephrine, and defibrillation, when needed (Table 3). End-tidal CO_2_ (ETCO_2_) and CPP are effective indices of resuscitation efforts.

**TABLE 3:** CPR parameters.

	Mechanical ventilation	Epinephrine	Chest compression	Defibrillation	Ventilation weaning
Keilhoff et al., 2017	FiO_2_ = 1.0	0.005 mg/kg, at initial	300/min	No	1 h post ROSC
Chang et al., 2020	FiO_2_ = 1.0	0.01 mg/kg, at initial and 0.02 mg/kg, every 2 min	200/min	No	1 h post ROSC
Hu et al., 2019	FiO_2_ = 1.0, a frequency of 80/min, tidal volume of 0.6 ml/100 g	0.02 mg/kg, at initial	240/min, with a depth of 25–30% of the anterior posterior diameter of the animal’s chest.	Single 2-J rectilinear biphasic shock	6 h post ROSC*
Zhu et al., 2018	FiO_2_ = 1.0	0.01 mg/kg, at initial	200/min	No	1 h post ROSC
Tae et al., 2017	FiO_2_ = 1.0	0.005 mg/kg, at initial	300/min	No	2 h post ROSC
Hansen et al., 2017	FiO_2_ = 1.0, a frequency of 105/min	0.01 mg/kg, every 2 min	200/min, with a depth of 1.4 cm (1/3 of anterior–posterior chest diameter) by mechanical chest compressions	No	Not mentioned
Keilhoff et al., 2017	FiO_2_ = 1.0, intermittent positive pressure ventilation	0.001 mg/kg, at initial	200/min	No	When sufficient spontaneous respiration was established

CPR parameters applied in several recent papers.

*Hu et al. (2019) study aimed to observe the effect of different post-resuscitation hyperoxia therapy durations, with ventilation weaning time extended to 6 h post-resuscitation.

CPR, cardiopulmonary resuscitation; ROSC, return of spontaneous circulation.

Standard chest compressions involve compression depth, frequency, and positioning. The literature indicates that chest compressions are performed at a rate of 200–300 times/min, at a depth of 1/3 the anteroposterior thorax diameter. Compressions can be performed manually, but maintaining uniformity of their frequency and depth is difficult, leading to interruptions. Electric chest compressors have therefore been invented in recent years. Compared with manual compressions, mechanical compressions can improve organ perfusion pressure, cerebral blood flow (CBF), and ETCO_2_ concentration (Vane et al., 2017). There are two chest compression approaches: cardiac pump and thoracic pump. Cardiac pump is performed by vertical sternum compression, thoracic pump by horizontal chest wall squeezing from both sides. The vast majority of experiments use cardiac pump. Okuma et al. (2019, 2021b) recently proposed a new approach: combining the two in a “three-side chest compression.” They found that this showed optimal CPR performance, improving CA survival. Its benefits may derive from increased intrathoracic pressure and stable cardiac hemodynamics. It may also improve reserve brain function. Yet three-side chest compression also increases operational difficulty.

Mechanical ventilation can improve early ACA outcomes and increase ROSC rates (Berg et al., 2000). The recent literature indicates that rat model mechanical ventilation is usually carried out with a small animal ventilator. Our procedure is to perform mechanical ventilation (FiO_2_ = 1.0) at the beginning of CPR at a frequency of 80–110 times/min with tidal volume 0.65 ml/kg. Although the optimal oxygen concentration during CPR is uncertain (O’Driscoll et al., 2017), current guidelines recommend maximum oxygen CPR to ameliorate tissue hypoxia (Newell et al., 2018). Clinical studies have shown that higher arterial oxygen partial pressure during CPR is associated with increased an ROSC rate (Spindelboeck et al., 2016; Demiselle et al., 2021).

Epinephrine, a systemic vasoconstrictor, can increase perfusion of important organs (i.e., the brain and heart) and is characterized by increased CPP (Putzer et al., 2020). The recommended epinephrine dose in adult advanced cardiac life support is 1 mg/3–5 min (Panchal et al., 2020). To model clinical practice, the dose used in rats ranges from 5 to 40 ug/kg, with 10 and 20 ug/kg commonly used. However, epinephrine can damage hemodynamics and cause myocardial injury. McCaul et al. (2006) studied the relations between epinephrine dose and outcomes in CA rats, showing that the group treated with 30 ug/kg had higher mortality than the 10 ug/kg group; that is, larger epinephrine dose was associated with increased mortality.

The 2020 CPR guidelines indicate that early defibrillation is beneficial for ROSC (Panchal et al., 2020). Several clinical studies have shown that VF in ACA is more common than previously thought (Herlitz et al., 2005a,b). In one ACA study in pigs, the initial rhythm converted from PEA to VF in 57% of pigs before CPR was begun (Varvarousi et al., 2015). If a shockable rhythm occurs, 2–4 joules (J) could be applied for defibrillation, with 2 J most commonly used in recent rat models (Hu et al., 2019; Yang et al., 2021). Electrical defibrillation can also have side effects, with too high defibrillation energy causing heart damage (Ishigaki et al., 2016). Xie et al. (1997) showed that in a CA rat model, increasing defibrillation energy from 2 to 20 J significantly reduced survival, cardiac index, and left ventricular function, possibly from abnormal intracellular Ca^2+^ kinetics (Ristagno et al., 2008).

ETCO_2_ is related to CBF (Lewis et al., 1992), and cardiac output and ETCO_2_ ware both affected by chest compressions (Gudipati et al., 1988). Thus, ETCO_2_ can be used for non-invasive monitoring of blood flow from chest compressions during resuscitation. In the 2020 American Heart Association guidelines, ETCO_2_ was recommended for assessing resuscitation quality (Panchal et al., 2020). Sustained increased ETCO_2_ (≥40 mmHg) is related to ROSC, and low or decreased ETCO_2_ suggests that CPR is low-quality. ETCO_2_ has also been associated with post-CA resuscitation outcomes in clinical trials. When ETCO_2_ < 10 mmHg, ROSC cannot occur (Sanders et al., 1989), the phenomenon also observed in rats. Abruptly increased ETCO_2_ is observed when ROSC occurs (Yagi et al., 2021), and remains higher in those who survive (20.1–16.3 mmHg) than in those who do not (2.0–6.0 mmHg) (Sato et al., 1993).

It is well known that a critical marginal myocardial blood flow level must be achieved for successful resuscitation (Ge et al., 2020). CPP is the difference between aorta and right atrial pressures. Increased CPP predicts increased myocardial blood flow and thus ROSC. Elevated CPP has also been associated with elevated ROSC in rat models (Wu et al., 2017; Wang et al., 2018), in which it is measured by inserting a catheter into the right ventricle (Wang et al., 2018), though use of this complex technology is infrequent.

**FIGURE 1:**
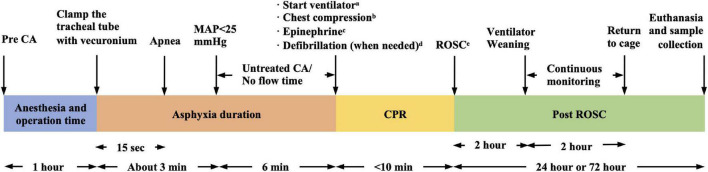
Six-min asphyxia cardiac arrest (ACA)/CPR model protocol (our protocol as an example). ^a^FiO_2_ = 1.0, frequency of 100/min, tidal volume of 0.65 ml/100 g. ^b^Manually, 200–300/min, at a depth of 1/3 the anterior–posterior chest diameter. ^c^0.01 mg/kg, every 2 min. ^d^Single 2J rectilinear biphasic shock. ^e^MAP > 60 mmHg lasting for ≥10 min. CA, cardiac arrest; CPR, cardiopulmonary resuscitation; ROSC, return of spontaneous circulation; MAP, mean arterial pressure.

The experimental ACA/CPR model protocol is shown in Figure 1. In conclusion, there are many factors to consider when building a CA model. Anesthesia drugs that interfere study purposes should be avoided. Analgesia should be considered in conjunction with sedation. For unskilled operators without advanced equipment, cervical exposure may be considered before endotracheal intubation. Combined with the neuromuscular blocking agent during asphyxia can reduce stress to the animal and control the duration of respiratory arrest. The duration of cardiac arrest is related to the severity of nerve damage and ROSC rate, so it is necessary to reasonably determine the CA duration in combination with the study purpose. ETCO_2_ and CPP are recommended to test the CPR efficiency.

## 4. Post-resuscitation management

Post-resuscitation oxygen therapy and hypothermia therapy are widely used in clinical practice, therefore, the inclusion of these two treatments in the animal model can better simulate the clinical situation.

### 4.1. Oxygen therapy

Cardiac arrest is a sudden cessation of circulation and respiration, with blocked oxygen delivery. Thus, oxygen support is needed during resuscitation and post-resuscitation to restore vitality, especially of the heart and brain. The oxygen concentration used with mechanical ventilation post-resuscitation should thus also be considered. Regarding hypoxic/ischemic disease, administration of hyperoxic gas may ensure vigorous resuscitation.

However, recent animal model findings have confirmed that hyperoxic ventilation post-resuscitation aggravates hypoxic/ischemic damage. Okuma et al. (2021a) studied post-resuscitation outcomes between normoxic (FiO_2_ = 0.3) and hyperoxic (FiO_2_ = 1.0) therapies in the ACA rat model, showing that the former reduced oxidative stress in multiple organs and improved organ injury, oxygen metabolism, and survival. Another ACA model study used target temperature management (TTM) in four hyperoxic ventilation groups (durations of 0, 1, 3, or 5 h) and a normoxic ventilation group (FiO_2_ = 0.21). The 3-h hyperoxic ventilation group had better neurologic outcomes and a higher survival rate compared with the normoxic ventilation group, while the other hyperoxic groups had worse outcomes (Hu et al., 2019). European guidelines from 2015 (Nolan et al., 2015) recommend maintaining blood oxygen saturation at 92–98% post-resuscitation, and have recently added an arterial oxygen pressure target of 10–13 kPa (75–100 mmHg) (Nolan et al., 2021b).

To conclude, applying oxygen therapy post-resuscitation is common in clinic, although the best oxygen therapy (including oxygen concentration and duration of oxygen therapy) has not been determined. But the current research shows that hyperoxic therapy at a specific time is better than normoxic therapy and long-term hyperoxia therapy. Additional studies are needed to explore optimal oxygen concentrations. Our protocol is to maintain hyperoxic ventilation (FiO_2_ = 1.0) for 1 h post-resuscitation, and normoxic ventilation (FiO_2_ = 0.21) for 1 h after that (Diao et al., 2020). We also withdraw mechanical ventilation 2 h post-resuscitation.

### 4.2. Hypothermia

In 2002, two prospective clinical studies of therapeutic hypothermia (TH) were published in the New England Journal of Medicine. Their results showed that mild hypothermia significantly improves survival and neurologic recovery after out-of-hospital CA compared with normothermia (Bernard et al., 2002; Hypothermia after Cardiac Arrest Study Group, 2002). These findings inspired researcher interest in this therapy, which has also been shown effective for preserving neurologic function. TH can inhibit activation of apoptosis, reduce post-injury inflammatory response, and produce neuroprotective effects (Hong et al., 2021). In addition, since the cerebral metabolic rate decreases 6–7% for every 1°C drop in body temperature (Kaylor et al., 2022), reducing oxygen demand may improve neurologic function.

Target temperature management is a broad concept that was first introduced in 2011; it includes TH, normal temperature control, and fever treatment (Nunnally et al., 2011). TTM is the only recommended post-resuscitation neuroprotective management, and is endorsed by both the American Heart Association and the Society of Critical Care Medicine (Kaylor et al., 2022). Animal experiments have repeatedly confirmed its neuroprotective effects (Hong et al., 2021; Wang et al., 2021b).

High-quality TTM requires several factors: initial timing, target temperature, cooling method, cooling duration, and rewarming duration. TTM should begin as soon as possible to shut down oxygen demand early. Most experts agree with a target temperature of 33–36°C. Low target temperatures theoretically provide better neurologic function but may also confer adverse events like unstable hemodynamics and hemorrhage. However, recent studies of patients with CA demonstrated that TTM at 33 or 36°C produces equivalent neuroprotective effects, while 36°C leads to fewer adverse events (Lee et al., 2018; Hong et al., 2021). Target temperature generally depends on patient condition and severity of neurologic damage. There are three main cooling methods: surface, intravenous-induced, and drug-induced. Surface cooling uses mechanical methods, like a cold room, ice blankets, icepacks, spraying the body with alcohol, and cold water baths, which require relatively long periods (∼30 min) to reach target body temperature (Yanamoto et al., 2001; Wang et al., 2010; Lagina et al., 2012). In contrast, intravenous-induced hypothermia, usually with an infusion of cold saline, achieves the target body temperature within 5 min (Wang et al., 2010). Recent hypothermia drug studies have shown that these can eliminate shivering by acting on central or peripheral thermoregulatory pathways, and can also be used to treat conscious patients with hypothermia (Lee et al., 2017a). In animal experiments, cold temperature durations range from 30 min to 6 h, with 4 h commonly used (Diao et al., 2020; Xu et al., 2022). Finally, rewarming rate is usually controlled at 0.5–2.0°C/h (Diao et al., 2020; Xu et al., 2022) or as the fastest rewarming speed (Jawad et al., 2021; Wang et al., 2022).

Hickey et al observed spontaneous hypothermia in the ACA model of rats (Katz et al., 1995, 1998; Hickey et al., 1996; Radovsky et al., 1997). And studies demonstrated that spontaneous hypothermia reduced neuronal damage and ameliorated inflammation and neurologic deficit (Hickey et al., 2000; Zhou et al., 2018). Therefore, maintaining normothermia is often needed to avoid variable temperature confounds. Rat’s normal core body temperature (rectal temperature) is about 37.5°C (Dangarembizi et al., 2017), therefore, most rat models control the normal body temperature at 37.5 ± 0.5°C (Tungalag et al., 2022; Xu et al., 2022). Heating pads or lamps can keep the rats at normal body temperature. During the operation, we should also pay attention to keeping the animals warm and keeping them away from the cold air. After the operation, the animals can maintain their body temperature in a constant greenhouse (Klahr et al., 2017). Many researchers are currently devoted to studying the neurologic protective effects of specific drugs combined with TTM post-resuscitation (Huang et al., 2016; Brucken et al., 2018; Nakayama et al., 2018). This area holds strong exploration potential.

Hypothermia therapy has been widely recognized in clinics. The setting of the target temperature needs to be considered in light of the neurologic deficit severity. In the experiments about hypothermia therapy, we should pay attention to the phenomenon of spontaneous hypothermia in animals, and guarantee heat preservation of the normal temperature control group.

## 5. Neurologic deficit assessment

It is important to assess neurologic deficits toward developing a successful model. Many methods exist for this purpose, including the NDS, cognitive function assessment, pathology, electroencephalogram (EEG), nerve injury biomarkers, magnetic resonance imaging (MRI), and microdialysis.

### 5.1. NDS

The NDS is used to evaluate neurologic deficit. Its template is patterned on the standard human neurologic examination. Different animals have corresponding scales; for rats, three are commonly used. First, Hendrickx et al. (1984) adapted a rat NDS from that used with monkeys. Second, Katz et al. (1995) developed a new NDS adapted from the canine version; this version consisted of five components: consciousness and respiration, cranial nerve function, motor function, sensory function, and coordination (including balance beam walking). Third, based on multiple animal model scales (including versions for the rat, canine, and neonatal piglet), Geocadin et al. developed a novel NDS in 2000. This version ranged from 80 (best) to 0 (brain dead) (Geocadin et al., 2000) and included seven parts: general behavioral deficit, brainstem function, motor assessment, sensory assessment, motor behavior, behavior, and seizures.

Rat neurologic function changes dynamically across the first few days post-resuscitation. Modi et al. (2022) used an NDS of 0–80 with a cutoff of 60 to distinguish mild and severe neurologic impairment. In their study, 24 h NDS scores were <60, which lowered at 48 h. That is, neurologic function deteriorated. In contrast, Zhang found that 24-h NDS was >60, with 48-h and 72-h scores gradually increasing and stable, indicating neurologic improvement. Thus we can assume that when the 24-h NDS is higher than the cutoff value neurologic function will gradually deteriorate, and that when the 24-h NDS is lower than the cutoff value neurologic function may recover gradually. Another ACA/CPR rat model study also showed stable NDS scores at 72 h and later (Chen et al., 2019). Therefore, 24 h is recommended as the early post-resuscitation neurologic function assessment timepoint, and 72 h or later should be used for long-term evaluation.

Though the NDS is a brief, convenient instrument for evaluating neurologic deficits, it is not without limitations. First, some items are unreasonable, like that proposed by Geocadin and Katz for which the only brainstem reflex options are “present” or “absent,” but may actually be “sluggish” in studies. In this respect, the NDS proposed by Hendrickx is superior. In addition, because the NDS is subjective, intra-investigator rating differences may lead to information bias, as may also occur if investigators are unblind regarding group assignment. Thus, it is recommended that at least two investigators rate on the NDS, both of whom should be blinded.

### 5.2. Cognitive function assessment

Cognitive function assessment is another aspect of neurologic deficit assessment. In the ACA rat model, we observed cognitive function impairment (Han et al., 2020). Animal behavioral assessments are often used to assess cognitive function. As for rodents, the maze test can evaluate memory and cognitive function. The Morris water maze (MWM) test assesses hippocampal-dependent learning, including the acquisition of spatial memory and long-term spatial memory. Studies have shown that at day 18 after resuscitation, ACA rats had significantly longer total swimming distances than shams before reaching the platform placed in the MWM test (Huang et al., 2019). The Y-maze test can evaluate spatial memory quantitatively and objectively. Studies have shown that in the Y-maze test, the spontaneous alternation rate decreased in rats’ post-resuscitation, suggesting cognitive impairment (Lee et al., 2020). Electrical stimulation is needed in Y-maze test, which will cause stress to rats. T-maze is analogous to the Y-maze, and can evaluate spatial memory quantitatively and objectively, but without electrical stimulation. In T-maze test, we also observed that the spontaneous replacement rate of ACA rats decreased (Lee et al., 2017b).

In addition to the maze test, the novel object recognition test can also assess cognitive function, performed based on the spontaneous tendency of a rat to explore a novel object. The higher recognition index (RI = novel object interaction time/total object interaction time) demonstrates a better cognitive function. Emulsified isoflurane postconditioning improves ACA rat’s neurological outcomes, characterized by elevated RI (Zhang et al., 2017).

However, it should be spotted that in CA rats with severe neurologic deficits, the behavioral tests mentioned above may not be completed for motor function damage. Therefore, we need to accurately evaluate the rats’ status to confirm whether behavioral experiments can be carried out.

### 5.3. Histopathology

Following CA, neuron and glial cell ultrastructure changes occur. Hematoxylin-eosin (HE) staining of perineuronal edema appears as dark neurons, soma, axons, and dendrites, especially among large neurons in the cortex and thalamus, clearly indicating large necrotic changes that may lead to cell death (Sharma et al., 2011). With inflammation, glial cells are activated and altered morphology or numbers are observed (Chang et al., 2020). Such pathology analyses can be used to observe cell-level nerve injury.

There are unique neural tissue methods in addition to conventional HE staining. Nissl staining clearly distinguishes Nissl bodies (which can be reduced or even eliminated with neuronal damage), nuclei, and nucleoli (Kiernan et al., 1998). Fluoro-Jade stains denatured neurons, which show blue fluorescence under ultraviolet light, allowing both qualitative and quantitative damage assessments (Schmued et al., 1997). Different glial cells have corresponding, specific staining methods. Cajal staining is a selective technique for astrocytes (Garcia-Marin et al., 2007), and immunohistochemistry or immunofluorescence analysis for glial fibrillary acidic protein (GFAP) can show the activation of astrocytes (Lu et al., 2022). Silver carbonate staining shows cell bodies and microglial processes (McCarter, 1940; Wang and Wei, 2012). Oligodendrocytes are stained with the del Rio Hortega method (McCarter, 1940). Neuron ultrastructures like cell membranes, cytoplasm, and organelles are visible with electron microscopy. In the 10-min rat CA model, researchers have observed clumping or condensation of neuronal chromatin, indented nuclei, and altered mitochondria and endoplasmic reticulum (Hossmann et al., 2001). Yasuda et al. studied the dynamic pathology changes of glial cells in transient global ischemia in rats (Yasuda et al., 2011). The results revealed the four phases of neuronal reduction post-resuscitation: (1) lag phase when very little neuronal loss was observed (day 0–2), (2) exponential phase when neuron reduced exponentially (day 2–7), (3) deceleration phase when the rate of reduction became lower (day 7–14), and (4) stationary phase when the additional neuronal loss was no longer observed (after day 14). From this point of view, it’s reasonable to evaluate the long-term neurological outcome in the deceleration phase or stationary phase. This seems to contradict the conclusion we reached in the “NDS” section. In fact, this study only assessed neuronal loss in the CA1 region of hippocampus, while NDS involves brain function in many regions. More research is needed to explore the relationship between NDS and neuronal reduction.

Human studies have shown that the hippocampus and basal ganglia are more sensitive to hypoxia/ischemia injury, which may serve as sentinels for post-CA ischemic pathology (Haglund et al., 2019). The degree of hypoxia/ischemia injury to the hippocampus and cerebellum is also slightly higher than to the cerebral cortex and thalamus (van Putten et al., 2019). Further, cortical, basal ganglia, and cerebellar slices are more time-consuming to prepare compared with hippocampal slices (Haglund et al., 2019). Thus, evaluating neurologic injury by hippocampal slices may be more feasible overall.

### 5.4. EEG monitoring

Following neurologic examination, EEG is commonly used to assess neurologic prognosis in hypoxic/ischemic encephalopathy. Through continuous, non-invasive bedside monitoring of neuronal electrical activity, with neurologic function assessment based on specific waveform characteristics, EEG plays an important dynamic monitoring role. Sustained isovoltage, low-voltage, or low-burst suppression patterns of EEG activity within the first 24 h predict a poor prognosis, whereas rapid return to continuous patterns within 12 h is strongly associated with better neurologic outcome (Hofmeijer and van Putten, 2016). Chen et al. (2019) used EEG to predict neurologic outcomes after resuscitation in hydrogen-treated rats, showing that this group had shorter EEG burst time and better neurologic prognosis compared with the control group.

However, the volume of information from conventional EEG precludes efficient interpretation. A solution to this issue, amplitude integrated EEG (aEEG) quantifies EEG temporal and spatial data. Alpha and beta rhythms decrease during ischemia in rats, while reperfusion promotes their recovery (Lu et al., 2001). At present, this method is mainly used to monitor neonatal neurologic function. More research is needed to explore aEEG manifestations in patients recovering from CA.

Rat EEG must be implanted and mounted *via* intracranial electrodes. Electrodes are placed with reference to the rat brain atlas and, distinct from the 16-lead EEG used in humans, 3–5 leads are used in rat models. The conventional method is to insert subdermal needles into the skull surface (Wang et al., 2021a) or implant screw electrodes *via* drilling (Shoaib et al., 2022). The traditional exposed intracranial electrode combined device is then embedded atop the rat head outside the scalp. However, traditional bare intracranial electrodes cause discomfort, influencing animal activity and eating, and interfering with experiments. Researchers have now developed intracranial electrodes that are implanted beneath the scalp, where they are better protected. Signals collected this way are consistent with the traditional method but do not affect animal food or water intake (Benovitski et al., 2022).

### 5.5. Biomarker—neuron-specific enolase

Biomarkers are simple to detect and commonly used in clinical practice for convenient monitoring. Likewise, in animal experiments, serum or plasma biomarkers are often used to detect and evaluate neurologic injury. Most commonly used, neuron-specific enolase (NSE), is the only guideline-recommended biomarker for prognosis of neurologic function in CPR (Sandroni et al., 2014). It is released by damaged neurons into circulation through the blood–brain barrier (Steinberg et al., 1983). Though continuously rising NSE is a strong predictor of poor neurologic function prognosis (Schrage et al., 2019), a cutoff value has yet to be established. In patients with CA, 72-h NSE level >33 ug/L (Youden index: 0.469) is associated with poor neurologic prognosis (Daubin et al., 2011).

Neuron-specific enolase is also widely used to monitor neurologic outcome post-CA in animal models. Rat model NSE release increases significantly after both CA and CPR (Qiu et al., 2017). Within the literature, NSE levels vary. Twenty-four hours post-resuscitation in an 8-min ACA rat model, Wang et al. (2020) found NSE levels of 0.35 ± 0.14 ng/ml, while Qiu et al. (2017) reported ∼6 ng/ml. NSE levels can even differ between experiments within the same report [e.g., post-resuscitation NSE was ∼100 and 10 ng/ml in successive experiments (Han et al., 2020)]. Thus, rat NSE levels may be dissimilar to those of humans, and the model’s normative threshold has yet to be determined. Nevertheless, rat NSE changes after neurologic damage, which can be used to indicate and evaluate neurologic deficit in research. NSE levels can confirm successful establishment of a CA model or test the effectiveness of an intervention (e.g., by testing for changes in NSE values between experimental and control groups).

Neuron-specific enolase has other limitations. It is also present in red blood cells, so hemolysis of blood samples may significantly affect NSE levels (Scolletta et al., 2012). Its reliability is also affected by sample storage and measurement methods (Rundgren et al., 2014).

### 5.6. MRI

Recently, MRI, especially diffusion weighted imaging (DWI), has become vital for predicting post-resuscitation neurologic injury. Compared with computed tomography, MRI is more sensitive to ischemic injury. During CA, ischemia and hypoxia lead to cell edema, which DWI can detect post-resuscitation. The apparent diffusion coefficient (ADC) value is an important quantitative index used to evaluate the extent and severity of cerebral ischemia injury (Solar et al., 2022). Decreased ADC values reflect diffusion of water molecules, indicating cell membraned damage (Norris et al., 2020). Wallin et al. (2018) performed post-CA MRI in 46 patients with acute hypoxic/ischemic injury, showing that reduced ADC values were more common in patients with poor prognosis. Animal experiments also increasingly apply MRI. Drabek et al. (2014) used arterial spin labeling MRI to show global and regional CBF differences between ACA- and VF-induced CA in rats. Wei et al. (2020) further explored the ability of MRI markers, showing that higher CBF and cerebral metabolic rate of oxygen (CMRO_2_) predict better early neurologic function post-resuscitation. Similar to clinical research, compared to sham, ADC is significantly lower in ACA rats post-resuscitation Liu et al., 2022).

### 5.7. Microdialysis

Microdialysis is a continuous intercellular fluid recording technology used to assess dynamic changes in biochemical mediums. Because extracellular fluid is the neuronal survival environment, it reflects changes in brain function and metabolism through biochemical substances like glucose, lactic acid, pyruvic acid, and glutamate (Hutchinson et al., 2015). Pyruvate is metabolized to lactate by anaerobic digestion during hypoxia, so the lactate to pyruvate ratio (L/P ratio) is used as a marker of this activity (Hlatky et al., 2004; Tisdall and Smith, 2006). Glutamate is another indicator of hypoxia/ischemia (Hlatky et al., 2004). Glycerol is considered a marker of hypoxia/ischemia and cell membrane rupture (Tisdall and Smith, 2006). Therefore, microdialysis can also be used in neurologic monitoring post-resuscitation, to quickly and accurately reflect brain metabolism changes. Clinical studies have shown that lactate and pyruvate levels increase post-resuscitation, and more so in those with cerebral performance category (CPC) scores from 3 to 5 than those with CPC scores from 1 to 2 (Molstrom et al., 2021). Microdialysis technology has also been applied in animal models (Pan et al., 2018; Putzer et al., 2018). In rat CA model, central nervous glucose decreases during CA, then increases significantly post-resuscitation. Moreover, L/P ratio and glutamate increase markedly post-resuscitation in a VF-induced CA model (Schober et al., 2016), consistent with assumptions. Microdialysis has also shown post-resuscitation neurologic outcome predictive accuracy. Further, a VF-induced CA rat model study showed that microdialysis can be used to discriminate between survivors and non-survivors 8 min post-CPR, and that non-survivors tend to have elevated brain glutamate levels (Hosmann et al., 2016).

### 5.8. Multimodality neural monitoring

**FIGURE 2:**
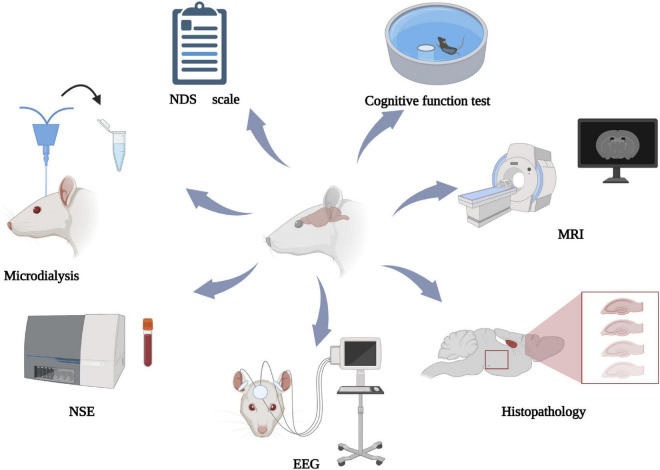
Multimodal neural monitoring diagram. NDS, neurologic deficit score; NSE, neuron-specific enolase; EEG, electroencephalogram; MRI, magnetic resonance. Created with BioRender.com.

The neurologic assessment methods mentioned above have their own advantages and disadvantages. NDS is simple and simple and easy to implement, but it lacks objectivity to some extent. Behavioral assessments need to be performed in rats with normal motor function. Histopathology can evaluate nerve function from a microscopic perspective, which is applicable to basic science research but not clinical practice. EEG and microdialysis can be used to analyze dynamic neurologic function. Serum biomarkers are easy to operate, but there is no unified standard detection method and cut-off value. Imaging examination is limited by the time limit of detection and cannot provide dynamic information. Not a single index can accurately evaluate neurologic function. Multimodality neural monitoring (MNM) is the combination of neural monitoring techniques (Riviello and Erklauer, 2021), including hemodynamic parameters and invasive and non-invasive methods described above. The 2021 guidelines of the European Resuscitation Council and European Society of Intensive Care Medicine emphasize use of combined methods to evaluate neurologic outcomes (Nolan et al., 2021a), as this approach is more accurate than single methods for guiding treatment (Anetakis et al., 2022; Huang et al., 2022). In animal experiments, MNM can also be used to accurately evaluate neurologic function and explore better joint prognostic models. Figure 2 illustrates MNM.

## 6. Conclusion

Cardiac arrest is a major public health issue, and the CA animal model is a good vehicle to pursue more knowledge about CA. Over recent decades, many researchers have been devoted to establishing an ACA rat model. The ultimate goal of animal research is to simulate clinical scenarios, to facilitate the successful transfer of research findings to clinical practice. For the complexity of cardiac arrest patients, no single animal model can perfectly represent them, therefore, designing models to meet different CA populations or scientific questions is needed. But these approaches must be transparent and reproducible to allow the investigators to judge the generalizability of the results.

The rats provide an excellent animal model of cardiovascular system and nervous system for its cost-based advantages and sufficient tissue for subsequent testing. Current CA models lack the post-resuscitation management and comorbidities which are needed to mimic clinical practice better. Oxygen therapy and hypothermia therapy are proposed as commonly used post-resuscitation management. It is suggested to consider post-resuscitation management measures when designing animal models. At present, animal models comorbid other related diseases are still scarce. It may be time to develop animal models with co-morbidities. The neurologic function is a vital indicator to evaluate whether the model is successful or whether interventions work. Common neurologic function assessments in animal experiments are reviewed, and it is suggested that researchers adopt a variety of methods to evaluate neurologic deficits in animal models.

A standard model establishment can help experiment homogenization. Although many factors must be considered in model construction, developing an ACA/CPR rat model is relatively simple and can meet experimental research needs.

## Author contributions

SY and CW performed the literature review and drafted the manuscript. YZ reviewed and edited the manuscript. WH and MD supervised, drafted, and revised the manuscript. All authors contributed to the manuscript and approved the submitted version.
